# Molecular and cellular adaptations to extended hypothermic oxygenated perfusion in donation-after-circulatory-death hearts in a porcine model

**DOI:** 10.3389/fcvm.2026.1800470

**Published:** 2026-05-25

**Authors:** Morgan K. Moroi, Yaagnik Kosuri, Cary Karcher, Diana Albino, Anthony Campbell, Arianna Adamo, Emre Bektik, Christine Chan, Kenmond Fung, Miroslav Sekulic, Shaheer K. Faruqi, Craig J. Goergen, Melissa Tamimi, Koji Takeda, Giovanni Ferrari

**Affiliations:** 1 Columbia University, New York, NY, United States; 2 University of Bologna, Bologna, Italy; 3 Ri.MED Foundation, Palermo, Italy; 4 Indiana University School of Medicine, Indianapolis, IN, United States; 5 Weldon School of Biomedical Engineering, Purdue University, West Lafayette, IN, United States

**Keywords:** bench-top reanimation circuit, cardiac preservation, donation after circulatory death (DCD), extended donor criteria, heart transplant, hypothermic oxygenated perfusion (HOPE), normothermic regional perfusion (NRP)

## Abstract

**Introduction:**

Donation after circulatory death (DCD) criteria serves as a potential strategy to expand the organ pool for heart transplantation. However, DCD procurement introduces prolonged warm ischemic times that accelerate endothelial and cardiomyocyte injury. Hypothermic oxygenated perfusion (HOPE) has emerged as a novel metabolic preservation strategy, yet its biological mechanisms remain undefined. We investigated how extended HOPE preservation modulates cardiomyocyte viability and metabolic stability after DCD in a porcine model.

**Methods and results:**

Following induced circulatory arrest and reperfusion with *in situ* normothermic regional perfusion (NRP), porcine hearts were preserved either by static cold storage (SCS) for 2 h or by HOPE for 24 h. A third experimental group included hearts that were directly procured without NRP and preserved by HOPE for 2 h. All hearts were subsequently reperfused and reanimated using an extracorporeal circuit under normothermic conditions. Flow cytometry revealed measurable populations of troponin-positive viable cardiomyocytes even after 24-hour HOPE, in contrast to complete loss after 24-hour SCS. RNA sequencing and metabolomics demonstrated minimal transcriptional or metabolic shift between 2-hour SCS and 24-hour HOPE hearts, with preservation of oxidative and glycolytic balance and limited inflammatory activation. Furthermore, omission of NRP during procurement resulted in marked loss of contractility and cardiomyocyte integrity, underscoring its potential role in pre-preservation harvest in a porcine model.

**Conclusions:**

In a swine model extended hypothermic oxygenated perfusion preservation sustains myocardial and metabolic integrity after circulatory death by minimizing transcriptional and metabolic injury signatures.

## Graphical Abstract

**Figure:**
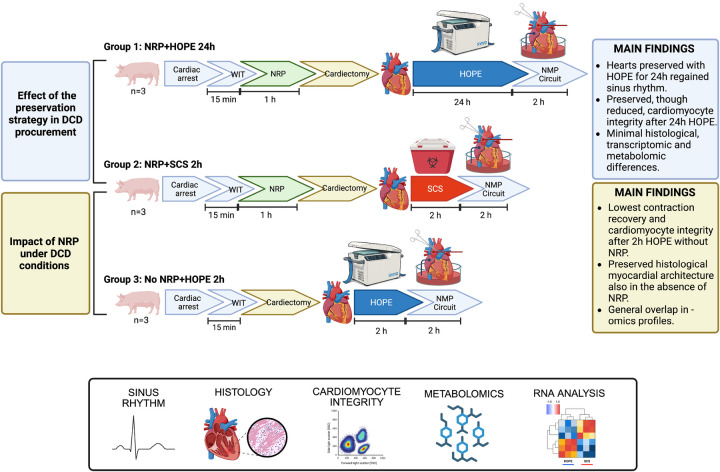

## Introduction

Heart transplantation remains the definitive therapy for end-stage heart failure, yet the availability of suitable donor organs continues to limit its clinical impact (1–3). Expanding donor criteria is one potential solution to address this shortage. While donation after brain death (DBD) remains the standard source of donor hearts, donation after circulatory death (DCD) has emerged as a promising but underutilized option (4–6). Two procurement strategies currently exist for harvesting DCD hearts in clinical practice. Both begin identically, with the withdrawal of life support, followed by circulatory arrest, confirmed by a standardized man arterial pressure (MAP) and asystole. Next, a center dependent, mandated provider stand off period, in minutes, is observed (7–9). In the first method, Direct Procurement (DP), emergent opening of the chest is followed by immediate delivery of cardioplegia before heart excision and preservation. Commonly, a normothermic machine perfusion (NMP) device, such as the Organ Care System (OCS) TransMedics Device is utilized in this approach (4). The second technique, adds thoracoabdominal normothermic regional perfusion (TA-NRP), referred to as NRP. This developing technique requires establishment of central cannulation, initiation of cardiopulmonary bypass, and cardiac reanimation *in situ*. Administration of this therapy affords providers a both functional evaluation of the allograft and reoxygenation of thoracic and abdominal organs prior to organ harvest (10). Recently, a group from Duke University transplanted a pediatric heart from a DCD donor using direct procurement with the inclusion of a bench-top NMP circuit (11). The NMP circuit allows for reperfusion and cardiac rhythm reanimation effectively replacing NRP in the heart harvesting process. However, the reanimated donor heart within the bench-top circuit is not physiologically loaded as in the case of traditional NRP, leaving behind uncertainty to this technique (12–15).

In addition to procurement strategies, preservation techniques also remain a critical barrier to organ sharing. Static cold storage (SCS) is used for cardiac allografts, but its upper limit is 4–6 h, and further prolonged ischemia directly correlates with primary graft dysfunction (PGD) (1, 16–18). Hypothermic oxygenated perfusion (HOPE) represents an alternative strategy for reducing ischemic injury and rejection (19).

XVIVO Heart Assist Transport, is a novel HOPE device being developed for this use. XVIVO's HOPE device has recently demonstrated encouraging results in clinical DBD trials across Europe, Australia/New Zealand and North America (19). In a recent study from our group, we reported that HOPE, under DBD conditions, enables 24 h *ex vivo* heart preservation with cardiomyocyte integrity, normal gross and microscopic architecture, and rapid functional recovery on bench-top reperfusion (20).

Given the growing interest in optimizing procurement strategies, improving preservation methods, and the limited mechanistic understanding of cardiac donation after circulatory death, this study aimed to evaluate both the contribution of extended HOPE preservation and NRP in DCD procurement. We compared preservation strategies following DCD procurement and *in situ* reanimation, evaluating hearts subjected to 60 min of NRP followed by either 2 h of SCS or 24 h of HOPE. Additionally, we assessed the impact of NRP by comparing DCD hearts preserved in SCS for 2 h following NRP, with DCD hearts preserved with 2 h of HOPE, which were procured via DP without NRP. This strategy allows for isolating and studying the effects of NRP as well as preservation technique in a porcine model.

## Methods

Key experimental procedures are described in the main Methods section, while additional protocol details are provided in Supplementary Materials and Methods section.

### Study design

This study evaluated extended hypothermic oxygenated perfusion (HOPE) as a preservation strategy for donation after circulatory death (DCD) hearts and examined the contribution of normothermic regional perfusion (NRP). A total of nine Yorkshire pigs were randomly assigned to one of the three experimental groups.

Group 1 (DCD + NRP + HOPE, *n* = 3): Animals underwent simulated DCD procurement with 15 min of warm ischemia (Supplementary Video S1), followed by 60 min of NRP (Supplementary Video S2), and subsequent preservation with HOPE for 24 h.

Group 2 (DCD + NRP + SCS, *n* = 3): Animals underwent simulated DCD procurement with 15 min of warm ischemia (Supplemental Video S1), followed by 60 min of NRP (Supplemental Video S2), and subsequent preservation with static cold storage (SCS) for 2 h.

Group 3 (DCD + direct procurement + HOPE, *n* = 3): Animals underwent simulated DCD procurement with 15 min of warm ischemia, followed by direct procurement (DP) without NRP, and preservation with HOPE for 2 h.

For the purpose of this study, two primary comparisons were performed:

− Preservation strategy comparison (Group 1 vs. Group 2): to evaluate the effect of SCS vs. prolonged HOPE following DCD + NRP.
− NRP contribution comparison (Group 2 vs. Group 3): to isolate the impact of NRP after short-term preservation.

All grafts were subsequently reanimated on a normothermic machine perfusion (NMP) circuit to simulate reperfusion injury. Study outcomes included cardiomyocyte viability, histological evaluation, transcriptomic profiling by RNA sequencing and metabolomic analysis, and a semi-quantitative functional assessment of ventricular wall velocity motion.

### Animal characteristics

Female American Yorkshire pigs (*n* = 9, 40–50 kg, 4–6 months old) were included in this study and randomly assigned to one experimental group. This study was approved by Columbia University (IACUC #AC-AABX9650) and performed under the NIH Guide for the Care and Use of Laboratory Animals.

### Simulating DCD procurement model

Swine donors underwent anesthesia with Telazol (21), propofol, and buprenorphine, were intubated, and maintained with isoflurane, midazolam, and propofol. After sternotomy, the great vessels were exposed, and systemic heparinization was achieved. For all groups, withdrawal of life sustaining therapy (WLST) was simulated by administration of a neuromuscular blocking agent, Vecuronium and cessation of ventilation. Functional warm ischemia (fWIT) was recorded as beginning at Mean Arterial Pressure (MAP) < 50 mmHg and ending with either cold cardioplegia flush or reperfusion by NRP. Time of circulatory death was declared when there was absence of visible arterial pulses on the arterial pressure tracing. Following circulatory death, a 15 min no-touch period was observed in all groups, and this was referred to as asystolic warm ischemia time (aWIT).

For groups requiring NRP (Groups 1 and 2, *n* = 6), venous, arterial, and cardioplegia cannulas were placed before commencing WLST. The aorta and right atrium were cannulated and connected to a circuit consisting of a venous reservoir with oxygenator (Affinity Fusion Oxygenation System, Medtronic, Minneapolis, MN), 3 T Heater-Cooler system (Liva Nova, London, UK), and centrifugal pump CP37 (Spectrum Medical, Gloucester, UK). Following the 15 min no-touch period, perfusion was re-established through NRP and maintained for 1 h before cardioplegic arrest and cardiectomy. During reperfusion, hearts were defibrillated as needed using internal paddles with 10-30J.

For Group 3, hearts (*n* = 3) were flushed with cold cardioplegia in their arrested state immediately following the 15 min no-touch period.

Although this order of proceedings would not occur in clinical DCD scenarios, for Groups 1 and 2, NRP cannulation was performed prior to WLST and a 15-minute standoff period was held in order to better standardize the DCD procurement model. In current human DCD procurements, the duration of function warm ischemic can vary considerably due to reasons such as variability in the time required for patients to progress to circulatory death, variability in timing of emergent sternotomy and subsequent NRP cannulation, etc. We therefore believed it was important to develop a DCD procurement model that standardized the timing of emergent sternotomy and cannulation, thereby mitigating variability in warm ischemic time as a potential confounder in our study and improving the reproducibility of our experiments. Similar DCD models in swine have been reported (21, 22).

### Preservation

Group 2 hearts (*n* = 3) were stored statically submerged in 1L of XVIVO Heart Solution at 4 °C for 2 h. Group 1 and 3 hearts were preserved by HOPE in the XVIVO Heart Assist Transport device for 24 h (*n* = 3) or 2 h (*n* = 3), respectively, at 8 °C, with an oxygenated (95% O₂/5% CO₂) perfusate. A 2 h storage time was chosen to represent a safe preservation window for both SCS and XVIVO. Utilizing this shorter time point enables direct comparison to a clinically accepted procurement approach, allowing assessment of non-inferiority of HOPE conditions relative to SCS. With the XVIVO Heart Assist Transport device, hearts were connected to the device via an aortic cannula and perfused with a root pressure of 20mmHg. The right and left atria remained widely open for drainage, and an LV vent was placed across the mitral valve. The perfusate was composed of 300–400 mL of donor packed RBCs mixed with XVIVO Heart Solution (2,500 mL) supplemented with XVIVO Heart Solution supplement, insulin, antibiotics, heparin, and potassium. Flow in the XVIVO device fluctuated from 140 to 40 mL/min to maintain the root pressure of 20mmHg. As perfusion progressed, the flow often decreased linearly with time. Heart weight was recorded after cardiectomy, at the end of preservation, and after 2 h of NMP.

### Bench-top normothermic machine perfusion (NMP)

After preservation, all hearts (*n* = 9) were reanimated on a custom NMP circuit for 2 h to model ischemia-reperfusion injury and assess rhythm and contractility under unloaded conditions. The circuit included a venous reservoir with Capiox FX05 oxygenator (Terumo Cardiovascular, Ann Arbor, MI), 3 T Heater-Cooler system (LivaNova, London, UK), and Quantum 6-in. roller pump (Spectrum Medical, Gloucester, UK). The NMP circuit was primed with porcine donor whole blood collected before arrest and heparin (2,000 units). Calcium gluconate and sodium bicarbonate were supplemented throughout based on blood gas results. Glucose was maintained above 70 mg/dL with dextrose supplementation as needed. Electrolyte disturbances were managed using zero-balance ultrafiltration (ZBUF) (DHF 06 Hemoconcentrator, Sorin, Livanova, London, UK). To initiate NMP, the aorta was cannulated and connected to the circuit. An LV vent was maintained, and the atria remained widely open. The circuit was slowly initiated, allowing the aorta to fill. Once fully de-aired, the aorta was cross-clamped to allow for coronary reperfusion. To recollect the drainage from the heart back to the pump, the heart was submerged in a collection reservoir in contact with the venous return line. The line pressure in the circuit was maintained at 200–300mmHg with circuit flow between 250 and 350 mL/min. Hearts were reperfused for 2 h and paced at 100bpm with epicardial pacing wires placed on the LV and RV. Internal paddles were used to defibrillate as necessary, utilizing 10J – 30J in the event of shockable arrythmia.

### Myocardial motion video quantification

A custom Python pipeline (Python 3.10; OpenCV, NumPy, SciPy) was used to quantify myocardial motion during NMP reanimation. Videos were calibrated using a pixel-to-centimeter scale, and a region of interest (ROI) was defined over the anterior ventricles. To correct for camera movement, frames were stabilized with dense optical flow before recomputing motion vectors. Velocity traces were derived from the mean vector magnitude within the ROI, converted to mm/s using calibration and frame rate, and smoothed with a Savitzky–Golay filter. Systolic and diastolic phases were tracked, and complete cardiac cycles were analyzed for motion quantification.

### Histological analysis

Endomyocardial biopsies from the right ventricular (RV) septum and left ventricular (LV) free wall were collected from all the hearts at the end of the normothermic reperfusion circuit. Samples were fixed in 10% formalin, paraffin-embedded, sectioned (5 µm), and stained with Hematoxylin and Eosin (H&E), Masson's trichrome, and Terminal deoxynucleotidyl transferase dUTP Nick End Labeling (TUNEL). For all experimental animals, two nonconsecutive sections from each biopsy were examined in blind by a clinical pathologist, who evaluated the presence of coagulative myocyte necrosis, inflammation, edema, hemorrhage, fibrosis, thrombosis, and apoptosis in three independent fields.

### Flow cytometry

RV septum biopsies (∼100 mg) were enzymatically digested filtered, and RBC-lysed. Cells were fixed, permeabilized, and stained with Alexa Fluor 647 anti-cardiac troponin T and DAPI. Cardiomyocytes were identified as troponin⁺/DAPI^+^ and gated by FSC/SSC to exclude debris and apoptotic bodies. Cardiomyocyte size and troponin expression were used as indicators of preserved cellular integrity and viability.

### RNA extraction and bulk RNA sequencing analysis

RNA was extracted from flash frozen apex biopsies obtained at the end of NMP reperfusion (RNeasy Mini Kit, Qiagen), quantified (Qubit 3.0 Fluorometer, Life Technologies) and quality-checked (Agilent TapeStation 4200, Agilent Technologies). Libraries were loaded on the Illumina NovaSeq instrument and sequenced (2 × 150 bp Paired End configuration). Reads were trimmed, aligned to the Sus Scrofa genome (STAR aligner), and counted (feature Counts from Subread package). Differential expression was performed with DESeq2 (padj < 0.05, |log₂FC| > 1). Results were visualized with volcano plots and heatmaps (DataMap), and KEGG pathway enrichment was assessed (ShinyGO).

### Untargeted metabolomic profiling

Global untargeted metabolomics was performed on flash-frozen left ventricular biopsies obtained at the end of NMP reperfusion, using UHPLC–high resolution accurate mass spectrometry. Metabolites were extracted by two-step homogenization in 1:3 methanol/water and 1:1 methanol/acetonitrile and analyzed using both hydrophilic interaction chromatography (HILIC) and reverse-phase separation in positive and negative ion modes, respectively. Feature annotation was achieved by an in-house reference library as well as HMDB, KEGG, LipidMAPS as databases. Differential metabolites were determined by Student's t-test and the Benjamini-Hochberg False Discovery Rate correction was applied to generate adjusted *p*-values. Metabolites were considered significantly altered if BH-adjusted *p*-value (FDR) < 0.05 and |log₂FC| > 0.693.

### Statistics

Cardiomyocyte integrity differences between groups were assessed using unpaired two-tailed Mann–Whitney U test, commonly used in cases of small sample size and when it is not possible to assess data normality. Data are expressed as mean ± SEM, and the results with *p* < 0.05 were considered significantly different. Gene expression levels were analyzed with the Wald test and the Benjamini–Hochberg correction was applied to generate adjusted *p*-values. Genes with an adjusted *p*-value < 0.05 and absolute log_2_ fold change value > 1 were called as differentially expressed genes. Significantly altered metabolites were evaluated using the Student's t-test, with *p*-values adjusted using the Benjamini and Hochberg FDR method, and the chosen thresholds for significance were adjusted *p*-value < 0.05 and absolute log_2_ fold change value > 0.693. Statistical analyses and data plotting were performed using GraphPad Prism (v10.2.0).

## Results

### Functional recovery of DCD + NRP hearts after SCS and HOPE preservation

**Figure 1:**
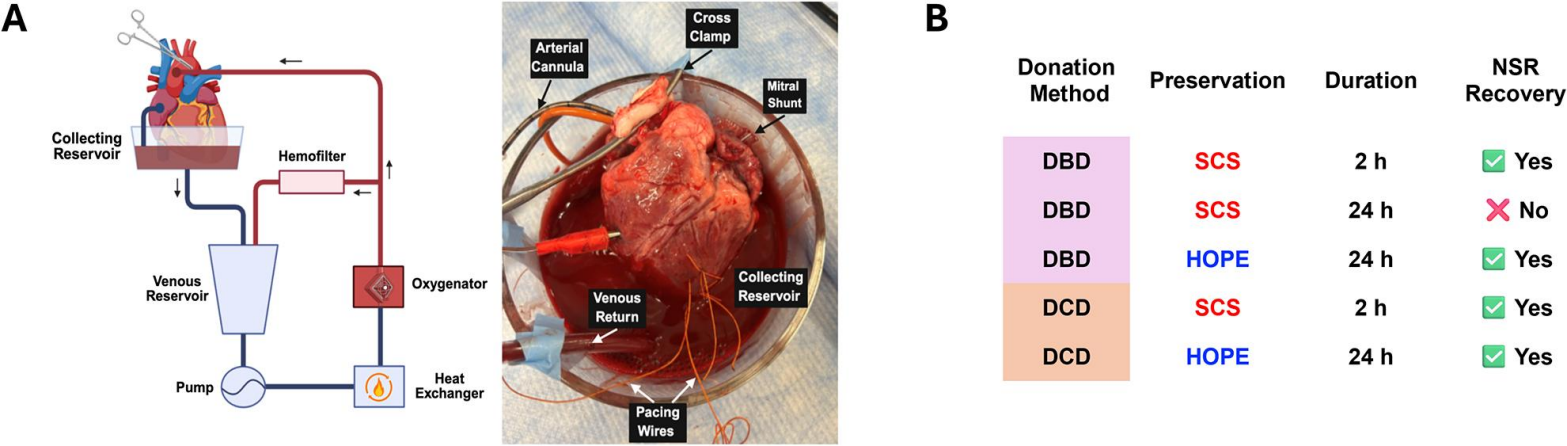
Functional recovery of DCD hearts following preservation and bench-top normothermic reperfusion. **(A)** Schematic and photograph of the normothermic machine perfusion (NMP) circuit. **(B)** Experimental groups and outcomes: DBD and DCD hearts preserved by SCS or HOPE for 2 h or 24 h. This table refers to data included in our previous work (20).

After hypothermic preservation, functional recovery of the hearts from Group 1 and 2 was assessed using a custom bench-top NMP system (Figure 1A). DCD + NRP hearts preserved by 2 h SCS (Group 2) consistently regained sinus rhythm and demonstrated robust contractile motion (Supplementary Video S3). Conversely, DCD + NRP hearts of Group 1, preserved by 24 h HOPE (three times the current clinical maximum duration) showed a reduction in contractile amplitude (Supplementary Video S4). Despite this reduction, all the DCD + NRP HOPE-preserved grafts successfully restarted, regained sinus rhythm, and maintained contractility exceeding pacer function throughout reperfusion (Figure 1B). Quantitative motion analysis confirmed significant differences in contractile velocity between Group 1 and 2. Average myocardial motion magnitude in the HOPE 24 h group was reduced compared with the SCS 2 h group indicating partial preservation of function (Supplementary Figure S1A, B).

### Gross examination and histopathological analysis

**Figure 2:**
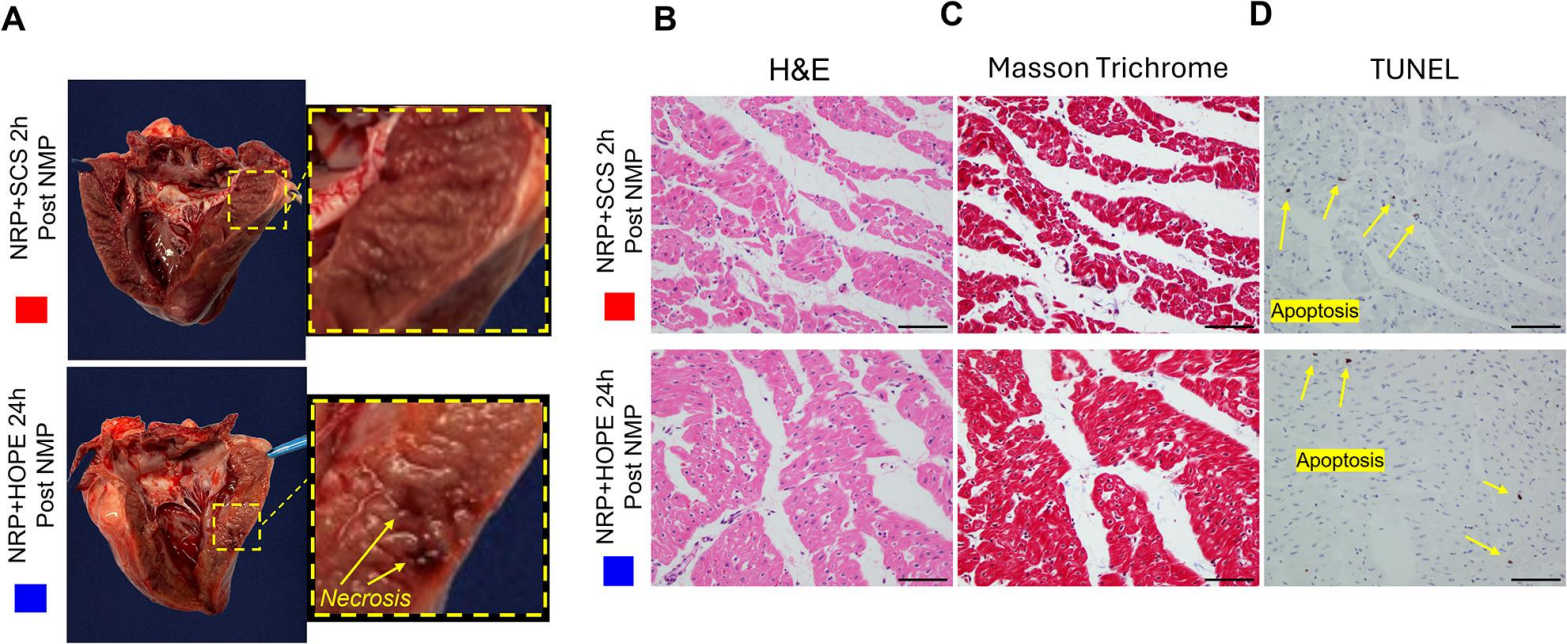
Gross morphology and histology analysis of swine hearts after NMP. **(A)** Hearts after reanimation by NMP following 2 h SCS (red) or 24 h HOPE (blue). Whole heart views with opened left ventricle show focal necrosis in 24 h HOPE, absent in 2 h SCS. **(B,C)** H&E and Masson Trichrome of the right ventricular septum show preserved architecture across groups. **(D)** TUNEL staining of the right ventricular septum shows comparable apoptotic cell positivity between groups. Scale bar: 100 µm.

Across groups, hearts gained weight during preservation and reperfusion, with HOPE-preserved grafts showing increases post-preservation, while all groups reached ∼15%–20% of baseline after NMP (Supplementary Figure S2). Following completion of the NMP circuit, ventricles were cut open; there was no evidence of necrosis in Group 2 hearts (NRP+2 h SCS). In contrast, trace of myocardial necrosis were visually evident in Group 1 (NRP+24 h HOPE) (Figure 2A). Histological evaluation was performed on endomyocardial biopsies collected from the RV and LV. Across all groups, H&E and Masson's trichrome staining showed preserved overall myocardial architecture without evidence of interstitial fibrosis or thrombosis. Consistent with our previous findings (20), edema was observed across all samples and was more pronounced in Group 1 hearts (Figures 2B,C; Supplementary Figure S3A). Coagulative myocyte necrosis (CMN) was very rare, identified in only one heart preserved in HOPE for 24 h. Inflammation was minimal, limited to focal neutrophil infiltrations in one 2 h SCS-preserved specimen. No cases demonstrated interstitial hemorrhage or vascular thrombosis. TUNEL staining revealed scattered apoptotic cardiomyocytes and interstitial cells in both Group 1 and Group 2 hearts, consistent with sub-lethal injury following ischemia-reperfusion (Figure 2D). However, no diffuse TUNEL positivity or confluent necrosis was observed, underscoring the absence of widespread irreversible cell death under either condition.

### Flow cytometric assessment of cardiomyocytes in DCD + NRP hearts

**Figure 3:**
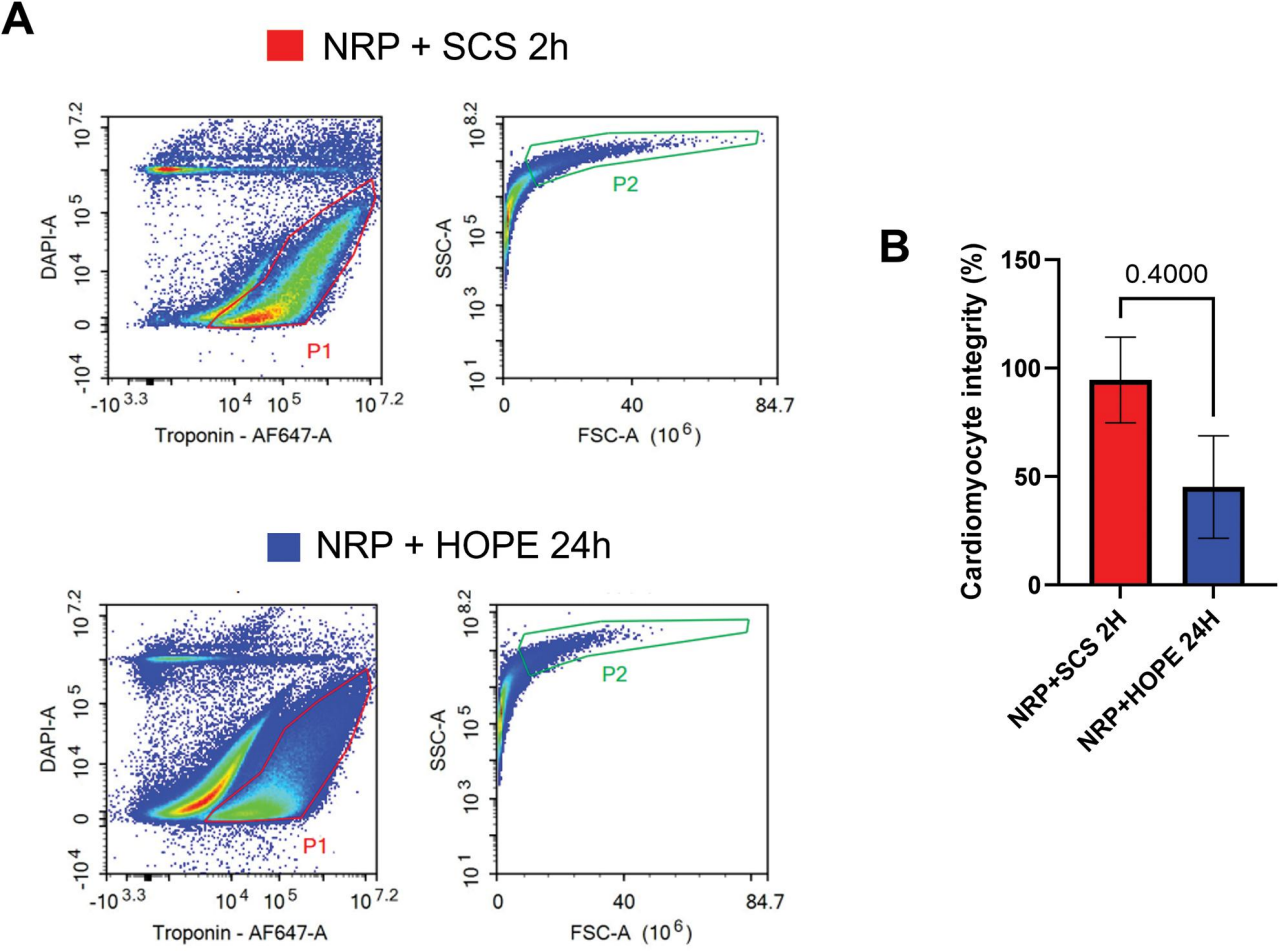
Cardiomyocyte integrity after extended HOPE preservation. **(A)** Flow cytometry of isolated cardiomyocytes after NRP + SCS 2 h (top) or NRP + HOPE 24 h (bottom). **(B)** Quantification of troponin⁺/DAPI⁺ cardiomyocytes (P2 gate) showed lower viability in NRP + HOPE 24 h vs. NRP + SCS 2 h. Data are presented as mean ± SEM and statistical significance was assessed using two-tailed Mann–Whitney U test (*n* = 3 animals per group).

Flow cytometric analysis was performed to quantify cardiomyocyte integrity following SCS and HOPE preservation. DAPI and anti-cardiac troponin T double-positive events (P1 gate) were further filtered by gating for SSC-A and FSC-A to exclude debris. In Group 2, isolated cardiomyocytes obtained after 2 h of SCS maintained cellular integrity and consequent viability (P2 gate). In contrast, in Group 1, extended preservation with HOPE reduced overall cellular integrity compared with 2 h SCS. However, a measurable population of cardiomyocytes was retained, indicating preserved cellular integrity despite prolonged storage and supporting the observed functional recovery (Figures 3A,B). Taken together, these findings align with functional and histological analyses, and prove that in a DCD model of procurement, HOPE preservation maintainsmeasurable cardiomyocyte structural integrity despite evidence of structural stress.

### Transcriptomic and metabolomics analysis on DCD + NRP hearts

**Figure 4:**
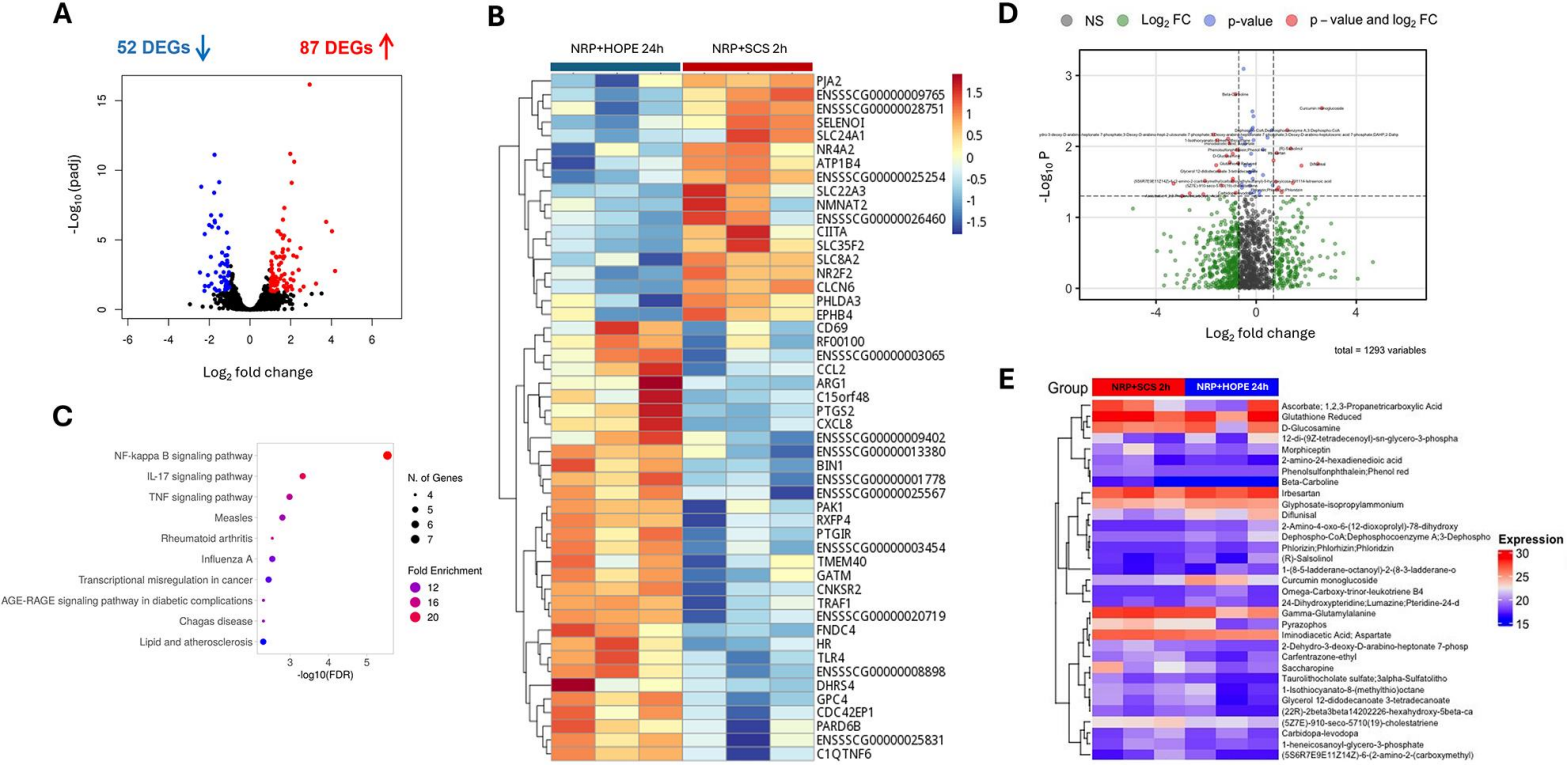
Transcriptomic and metabolomic profiling of DCD hearts. **(A)** Volcano plot of differentially expressed genes (DEGs) between NRP + HOPE 24 h and NRP + SCS 2 h (87 upregulated, 52 downregulated genes in NRP + HOPE 24 h; *p* < 0.05, |log_2_FC| > 1). **(B)** Heatmap of top 50 DEGs shows distinct expression patterns. **(C)** KEGG enrichment of upregulated DEGs highlights inflammation-related pathways (FDR < 0.01). Downregulated DEGs showed no significantly enriched pathways. **(D)** Volcano plot of metabolomic analysis shows significantly altered metabolites (red) (*p* < 0.05, |log₂FC| > 0.693). **(E)** Supervised clustering heatmap of 33 significantly changed metabolites shows no consistent shifts in central metabolic networks (*n* = 3 animals per group).

To investigate the cellular and molecular consequences of extended hypothermic oxygenated preservation, transcriptomic and metabolomic profiling was performed on DCD hearts from Groups 1 and 2. RNA sequencing revealed minimal transcriptional differences between groups. Differential gene expression analysis identified only a limited number of genes (87 upregulated and 52 downregulated in NRP + HOPE 24 h) meeting statistical significance (*p* < 0.05, |log_2_FC| > 1), with limited enrichment of gene ontology terms across biological processes, molecular functions, or cellular components. Pathway-level interrogation confirmed the absence of coordinated transcriptional shifts, indicating that extended HOPE preservation maintained a transcriptomic state broadly comparable to short-term SCS (Figures 4A,B; Supplementary Figure S5A–C). KEGG pathway enrichment analyses of upregulated DEGs in Group 1 hearts show that mostly inflammation-related signaling reached statistical significance (FDR < 0.01) while KEGG pathways for downregulated DEGs did not reach statistical significance (Figure 4C). Data are accessible at GEO #[GSE316371]. Metabolomic profiling demonstrated molecular stability across preservation strategies. While 33 metabolites were found to be differentially abundant (*p* < 0.05, |log_2_FC| > 0.693), pathway analysis revealed no consistent alterations in core metabolic networks. Global clustering of annotated metabolites showed substantial overlap between the two groups, underscoring the preservation of metabolic homeostasis during prolonged HOPE storage (Figures 4D,E; Supplementary Table S1). Data are accessible on Metabolomics Workbench #[DOI: http://dx.doi.org/10.21228/M8BC49].

### Functional recovery, cardiomyocyte integrity and histopathological analysis in NRP contribution study

**Figure 5:**
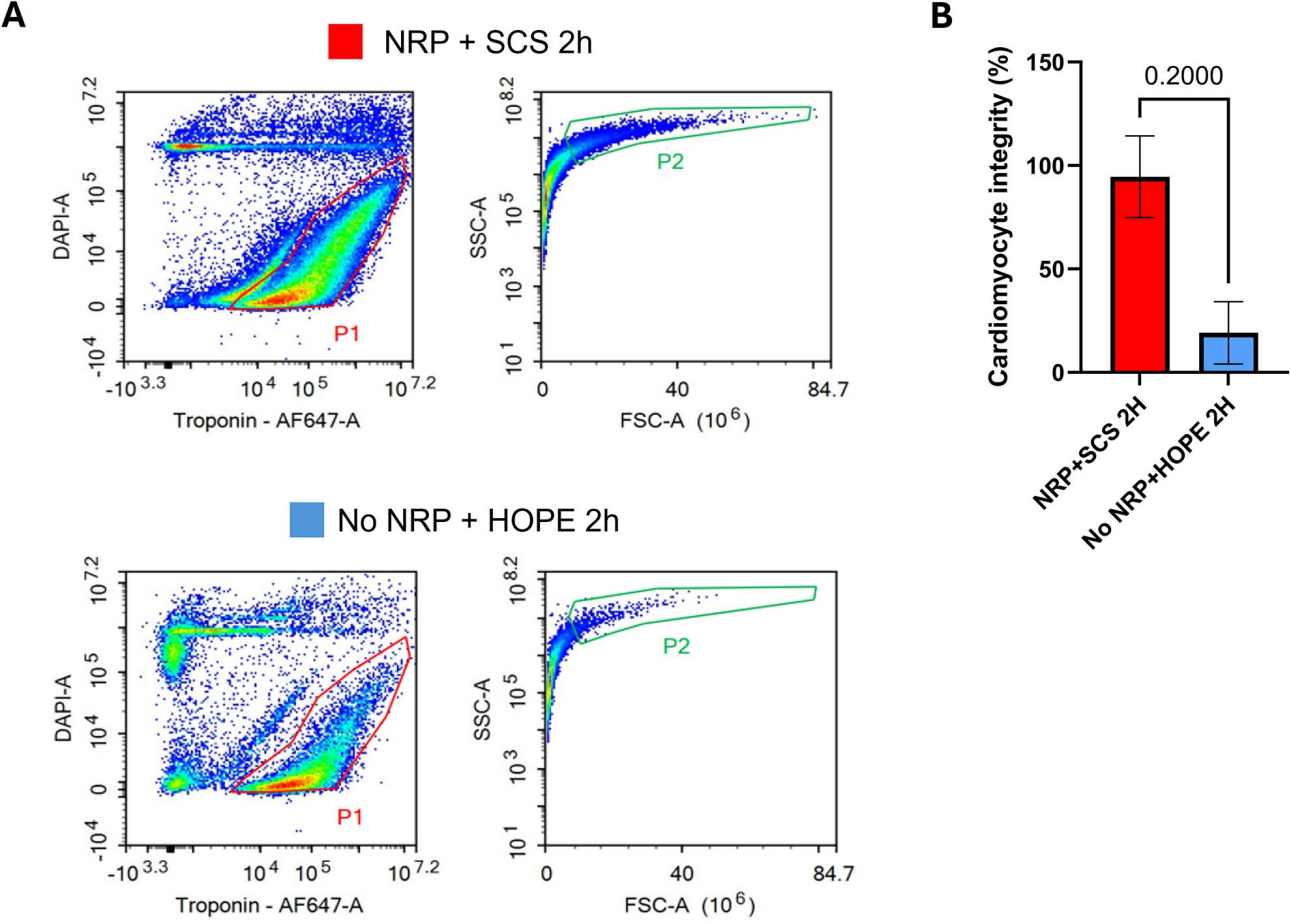
Impact of NRP in DCD hearts procurement: cardiomyocyte integrity. **(A)** Flow cytometry plots of troponin/DAPI staining show greater viability in NRP + SCS 2 h. **(B)** Quantification of troponin⁺/DAPI⁺ cardiomyocytes (P2 gate) confirmed higher viability in NRP + SCS 2 h. Data are presented as mean ± SEM and statistical significance was assessed using two-tailed Mann–Whitney U test (*n* = 3 animals per group).

To determine whether *in situ* reanimation with NRP influences porcine heart physiology during DCD procurement, we compared hearts from Group 2 (NRP + SCS 2 h), which were well within the clinical standard of preservation (1), with hearts from Group 3, which were directly procured without NRP administration and subsequentially preserved for 2 h in HOPE. All grafts were subsequently reanimated on a NMP circuit. Hearts procured without NRP (Group 3) exhibited the most severely impaired functional recovery, with depressed myocardial motion and reduced ability to sustain contractility above intrinsic pacer function (Supplementary Figure S1C, D; Supplementary Video S6). Assessment of cardiomyocyte integrity confirmed these findings. Hearts from Group 3, preserved with HOPE 2 h in the absence of NRP, displayed the lowest proportion of troponin-positive/DAPI-positive cardiomyocytes compared with both Group 1 and Group 2. (Figures 5A,B). These results suggest that the absence of NRP has a detrimental effect on the viability of myocardial tissue in DCD-procured hearts and that NRP may be a critical step for stabilizing DCD hearts prior to cardioplegia delivery and preservation in a porcine model. The histological evaluation of endomyocardial biopsies stained with H&E and Masson Trichrome revealed preserved general myocardial architecture of the right ventricular septum and left ventricular free wall. Edema was consistently observed in all samples, while no signs of fibrosis, thrombosis, interstitial hemorrhage and coagulative myocyte necrosis were reported. Fragmented DNA was identified by TUNEL staining in scattered cardiomyocytes and interstitial cells, though not consistent with a diffuse apoptotic pattern. (Supplementary Figure S3A, B).

### Transcriptomic and metabolomics analysis in NRP contribution study

**Figure 6:**
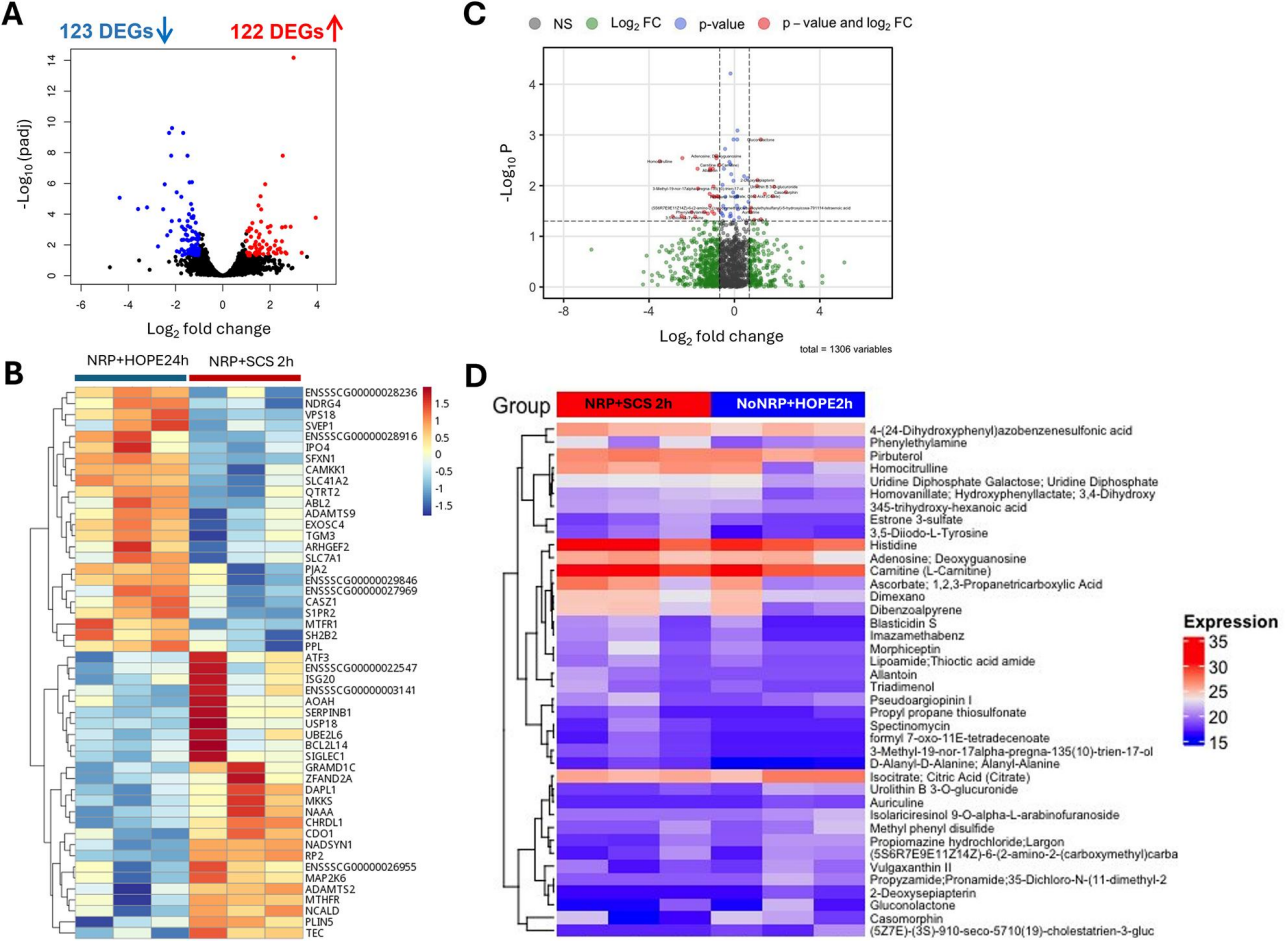
Transcriptomic and metabolomic profiling of DCD hearts preserved with NRP + SCS (2 h) or No NRP + HOPE (2 h). **(A)** Volcano plot shows 245 differentially expressed genes (122 upregulated, 123 downregulated genes in No NRP + HOPE 2 h; *p* < 0.05, |log_2_FC| > 1). **(B)** Heatmap of the top 50 DEGs demonstrates distinct transcriptional patterns. Neither up nor down-regulated genes showed any significantly enriched pathways. **(C)** Volcano plot of metabolomics highlights significantly altered metabolites (red). **(D)** Supervised clustering heatmap of top 40 significantly changed metabolites (*p* < 0.05, |log₂FC| > 0.693) shows group separation without consistent pathway-level enrichment (*n* = 3 animals per group).

Bulk RNA sequencing revealed minimal differences between hearts in Group 2 and 3. Although a limited number of individual genes met nominal significance (122 upregulated and 123 downregulated genes in Group 3), KEGG pathway analysis failed to identify consistent changes (FDR < 0.01). Unsupervised clustering confirmed substantial overlap in the global transcriptomic profiles, indicating that surviving cardiomyocytes retained comparable transcriptional signatures regardless of NRP exposure (Figures 6A,B; Supplementary Figure S5D–F). Similarly, metabolomic profiling identified 40 differentially regulated compounds (*p* < 0.05, |log_2_FC| > 0.693), but these differences did not converge into coherent shifts in central energy metabolism, amino acid pathways, or oxidative stress networks (Supplementary Table S2). Both supervised and unsupervised clustering analyses showed significant overlap between groups, suggesting metabolic stability independent of procurement strategy (Figures 6C,D). Taken together, these findings indicate that the benefits of NRP observed at the cellular and functional levels are not explained by broad transcriptional or metabolic reprogramming. Data are accessible at GEO #[GSE316371] and Metabolomics Workbench #[DOI: http://dx.doi.org/10.21228/M8BC49].

## Discussion

DCD represents an important yet underutilized source of cardiac grafts that could substantially expand the donor pool. The primary concern relates to the unavoidable exposure of the myocardium to warm ischemia at the time of circulatory arrest, which rapidly initiates profound metabolic derangement, cellular dysfunction, and structural injury. The ischemic damage raises legitimate questions regarding myocardial integrity and functional recoverability. In this context, defining the role of NRP is critical to understanding how early metabolic resuscitation may condition the heart prior to ex situ preservation and ultimately influence graft viability. Moreover, investigating preservation strategies capable of actively mitigating ischemia/reperfusion injury, like extended HOPE, is of great relevance.

In this study, we used a porcine model to elucidate the biological mechanisms through which HOPE preserves myocardial integrity after circulatory death and to define the contribution of NRP in supporting subsequent functional recovery. Overall, our results demonstrate that HOPE markedly extends the duration of myocardial preservation while maintaining cellular and metabolic stability. Additionally, NRP serves as a critical preconditioning step to enable recovery after circulatory arrest and evaluate of grafts before acceptance.

During the 60 min of NRP prior to DCD procurement, normal sinus rhythm and heart contractility was restored, likely through the replenishment of ATP and the washout of ischemia related metabolites. All the hearts preserved with HOPE for up to 24 h (Group 1) resumed sinus rhythm during NMP (Supplementary Figure S1A, B; Supplementary Video S4). This preserved capacity for cardiac reanimation after 24 h of HOPE is particularly relevant when compared to our previous study, in which hearts procured under favorable DBD conditions failed to reanimate after an equally long period (24 h) of SCS preservation (Figure 1B; Supplementary Video S5) (20). Despite preserved overall contractile performance of Group 1 hearts, the gross examination revealed focal areas of visually evident myocardial necrosis (Figure 2A). This outcome highlights a critical difference with our previous DBD work, in which hearts preserved for 24 h using HOPE did not show macroscopic signs of necrosis (20). Importantly, the histological analysis did not show diffuse TUNEL positivity or confluent necrosis, underscoring the absence of widespread irreversible myocardial injury (Figure 2D). Consistent with our previous observations (20), myocardial edema was present across all samples and was more pronounced in hearts from Group 1 (Figures 2B,C; Supplementary Figure S3A). Flow cytometric assessment of troponin^+^/DAPI^+^ cardiomyocytes fraction revealed a reduction in Group 1 compared with the short-term (2 h) SCS preservation (Figures 3A,B) in Group 2. However, the retention of a measurable cardiomyocyte population along with the persistence of synchronized electrical activity after 24 h highlights the capacity of HOPE to maintain essential metabolic and structural programs beyond conventional ischemic limits, even within the challenging DCD model. Conversely, in our previous study, we demonstrated that 24 h of storage in SCS failed to preserve cellular integrity even under more favorable DBD heart procurement conditions; no intact cardiomyocyte population was detected after the same flow cytometric assessment, consistent with irreversible cellular injury (Supplementary Figure S4) (20).

At the molecular level, both transcriptomic and metabolomic profiling revealed striking stability under HOPE conditions. Despite extended preservation, global gene expression and metabolic signatures remained comparable to 2 h SCS, suggesting suppression of injury-related signaling and maintenance of oxidative and glycolytic balance. Limited enrichment of inflammatory and stress-response pathways further supports the concept that HOPE provides a nearly quiescent metabolic state that mitigates ischemia/reperfusion–induced transcriptional activation (Figure 4). The preservation of redox homeostasis and mitochondrial function under hypothermic oxygenation likely contributes to the sustained viability observed in flow cytometry and functional assays. These findings align with prior observations that oxygenated hypothermia limits succinate accumulation and reactive oxygen species generation during reperfusion, hallmarks of improved mitochondrial stability (23).

Finally, we assessed the impact of NRP in a porcine DCD model by comparing hearts procured without NRP and preserved with 2 h of HOPE (Group 3) to those procured with NRP and preserved with 2 h of SCS (Group 2). Findings from the first part of this study, together with our previous work (20), support the non-inferiority of extended HOPE preservation relative to short-term SCS. Accordingly, we would not expect a meaningful difference between HOPE and SCS over a short 2-hour preservation window, allowing the observed differences to be attributed primarily to the presence or absence of NRP. The omission of NRP prior to procurement produced the most profound loss of contractile recovery (Supplementary Figure S1C, D; Supplementary Video S6) and cellular integrity (Figures 5C,D). Although bulk RNA sequencing and metabolomics detected few differences between NRP and no-NRP hearts from Groups 2 and 3, this likely reflects selective survival of metabolically competent cells and the limitation of bulk analyses to detect region-specific or endothelial injury (Figure 6). Functionally, NRP appears to provide an essential bridge between ischemic arrest and cold preservation, stabilizing mitochondrial metabolism and reducing microvascular obstruction through washout of prothrombotic and inflammatory mediators.

Collectively, these data define a two-step protective paradigm in DCD heart preservation. NRP re-establishes metabolic competence and vascular perfusion following warm ischemia, while HOPE maintains this quiescent, oxygenated state over extended durations by preventing energetic collapse and transcriptomic drift. This combined approach may represent a mechanistically grounded platform for mitigating ischemia/reperfusion injury, with implications extending beyond cardiac transplantation to the broader context of organ preservation, limitation of myocardial ischemia, and endothelial protection.

Several limitations should be acknowledged. First, *ex vivo* bench-top reanimation cannot fully replicate *in vivo* loading or immune responses. The small sample size reflects the logistical, economical and ethical complexity of large-animal models. Despite the low sample size, our results were highly consistent across animals within each group, and given the exploratory nature of the study, the number of pigs was considered sufficient to detect significant biological differences. Similar works on swine DCD procurement models also refer to small sample groups, supporting our methodology (21, 24). Additionally, bulk transcriptomic and metabolomic profiling capture only surviving cellular populations and may underestimate sublethal or spatially restricted injury. Future studies integrating single-cell analyses and endothelial-specific readouts will help delineate the vascular contribution to hypothermic oxygenated protection. Finally, the myocardial motion video quantification included in this study is a semi-quantitative method used to objectively assess contractility during bench-top normothermic machine perfusion. Of note, video acquisition was not standardized across all analyzed samples, representing a limitation given that cardiac contractility frequently improved throughout the 2 h NMP circuit duration. Furthermore, motion quantification was performed on one video per group, and therefore these findings should be considered preliminary. Nevertheless, these data further support the molecular and cellular findings of this study. An additional limitation to this study relates to our DCD model. Given the well-established relationship between cardiac injury and warm ischemic time, we sought to develop a DCD procurement model that minimized variability in the exposure to warm ischemia. Accordingly, heparinization and cannulation for NRP were performed prior to the initiation of circulatory death, and a 15-min stand-off period was incorporated to simulate, in a controlled fashion, both the donor center stand-off interval and the time typically required for emergent sternotomy and cannulation. We recognize this approach differs from the standard clinical practice of DCD procurement using TA-NRP, in which the duration of cardiac warm ischemia can vary significantly based upon surgeon and donor center protocols. However, this methodology was intentionally employed to minimize variability in warm ischemic time and reduce its impact as a potential confounder in our study.

In conclusion, hypothermic oxygenated perfusion establishes a biologically distinct preservation state that maintains metabolic, transcriptional, and structural stability after circulatory death. When preceded by normothermic regional perfusion, this approach minimizes ischemia/reperfusion injury and sustains myocardial viability far beyond the conventional temporal limits of static preservation. These findings identify mechanistic targets, such as mitochondrial stabilization, endothelial integrity, and redox balance, that may inform novel interventions to prevent ischemic injury across cardiovascular and organ preservation contexts (25, 26).

## Data Availability

The datasets presented in this study can be found in online repositories. The names of the repository/repositories and accession number(s) can be found below: https://www.ncbi.nlm.nih.gov/geo/, GSE316371 https://www.metabolomicsworkbench.org, http://dx.doi.org/10.21228/M8BC49.

## Supplementary material

Supplementary Table S1 Differentially expressed metabolites between NRP + SCS 2h and NRP + HOPE 24 h hearts associated to the relative functional pathways.

Supplementary Figure S1 Myocardial motion video quantification. Velocity traces during bench-top reperfusion show greater contractile amplitude in NRP+SCS 2 h vs NRP+HOPE 24 h **(A)** and vs No NRP+HOPE 2 h **(C)**. Quantification of average velocity magnitude (mm/s) ± SEM confirmed higher contractile activity in NRP+SCS 2 h for both comparisons (p < 0.0001 by unpaired two-tailed Student's t-test followed by two-tailed Mann–Whitney U test) (**B** and **D**).

Supplementary Figure S2 Heart weight changes during preservation and reperfusion. Heart weights were measured at baseline, after preservation, and following 2 h of normothermic machine perfusion (NMP). Both NRP+SCS (2 h) and NRP+HOPE (24 h) hearts showed comparable total weight gain (∼20%), while No NRP+HOPE (2 h) hearts exhibited a smaller increase (∼15%). Data are shown as mean ± SEM. Two-way Anova analysis did not show any statistically significant differences.

Supplementary Figure S3 Histological analysis of cardiac tissue. **(A)** Hematoxylin and Eosin (up) and Masson Trichrome(down) staining of the Left Ventricular Free Wall obtained by endomyocardial biopsies. **(B)** H&E, Masson Trichrome and TUNEL staining of RV Septum endomyocardial biopsies obtained from a No NRP heart preserved in HOPE for 2 h. Scale bar: 100 µm.

Supplementary Figure S4 Cardiomyocyte integrity after extended SCS preservation in a model of Donation following Brain Death (DBD). **(A)** Representative flow cytometry plots of DAPI and troponin staining in isolated cardiomyocytes after DBD procurement followed by 24 h SCS and 2 h of NMP reanimation circuit. Cardiomyocytes were identified as DAPI-positive, cardiac troponin T- positive events (P1 gate) and further selected for intact cells in a FSC vs SSC plot (P2 gate). The population of isolated intact cardiomyocytes was severely depleted, with no preserved cellular viability following 24 h of SCS, despite the more favorable donation model (20).

Supplementary Figure S5 Transcriptomic profiles of DCD hearts preserved with NRP+SCS 2 h versus HOPE 24 h or No NRP+HOPE 2 h. **(A–C)** Comparison of NRP+SCS 2 h and NRP+HOPE 24 h **(A)** Principal component analysis (PCA) shows partial separation between groups. **(B)** Boxplots of normalized read counts illustrate similar global expression distributions across samples. **(C)** Unsupervised hierarchical clustering heatmap of the top differentially expressed genes demonstrates partial grouping by preservation strategy. **(D–F)** Comparison of NRP+SCS 2 h and No NRP+HOPE 2 h. **(D)** PCA again shows modest separation between groups. **(E)** Boxplots of normalized read counts show comparable expression distributions. **(F)** Unsupervised clustering heatmap reveals overlap between groups.

Supplementary Table S2 Differentially expressed metabolites between NRP+SCS 2 h and No NRP+HOPE 2 h hearts associated to the relative functional pathways.

Supplementary Video S1 Circulatory arrest swine open-chest

Supplementary Video S2 Normothermic regional perfusion

Supplementary Video S3 Bench-Top NMP Reanimation after NRP + SCS 2h

Supplementary Video S4 Bench-Top NMP Reanimation after NRP+HOPE 24h

Supplementary Video S5 Bench-Top NMP Reanimation after DBD+SCS 24h

Supplementary Video S6 Bench-Top NMP Reanimation after No NRP+HOPE 2h
