# Supplementary material for: Molecular and cellular adaptations to extended hypothermic oxygenated perfusion in donation-after-circulatory-death hearts in a porcine model
Source: Front Cardiovasc Med. 2026 May 25;13:1800470. doi: 10.3389/fcvm.2026.1800470 (PMC13243425; doi:10.3389/fcvm.2026.1800470)
Supplement: Supplementary Table S1 — Differentially expressed metabolites between NRP + SCS 2h and NRP + HOPE 24 h hearts associated to the relative functional pathways. [file Table1.docx]

**Supplemental Tables.**

| **PATHWAY** | **METABOLITE** | **log_2_FC** | **p-value** | **FDR** |
| --- | --- | --- | --- | --- |
| Glycolysis and Gluconeogenesis | L-Lactic Acid (Lactate) | 0.269427628 | 0.011055465 | 0.688766538 |
|  | Hexoses | 0.123270327 | 0.145066969 | 0.752052236 |
|  | Lipoamide | -1.158899985 | 0.589668745 | 0.896869129 |
|  | | | | |
| TCA Cycle | Oxoglutaric Acid (Oxoglutarate) | -0.143588028 | 0.122193562 | 0.752052236 |
|  | L-Malic Acid; D-Malic Acid (Malate) | -0.160439044 | 0.159799752 | 0.752052236 |
|  | | | | |
| De novo fatty acid biosynthesis | Palmitic Acid (Palmitate) | -0.109739746 | 0.003755526 | 0.661896577 |
|  | Eicosatrienoic Acid | 0.131403798 | 0.043511251 | 0.752052236 |
|  | Lauric Acid (Laurate) | 0.197498505 | 0.159722904 | 0.752052236 |
|  | | | | |
| Glycine, serine, alanine and threonine metabolism | Ornithine (L-Ornithine) | -0.820203329 | 0.061469568 | 0.752052236 |
|  | Aminobutyric Acid | -0.389490149 | 0.079646235 | 0.752052236 |
|  | Methionine | -0.876231019 | 0.10061039 | 0.752052236 |
|  | Propionylglycine | -0.62663847 | 0.102887738 | 0.752052236 |
|  | Glyceric acid | -0.274318603 | 0.117323995 | 0.752052236 |
|  | Oxoglutarate (Oxoglutaric Acid) | -0.143588028 | 0.122193562 | 0.752052236 |
|  | Homoserine; Allothreonine | -0.136531272 | 0.123276803 | 0.752052236 |
|  | Threonine | -0.136531272 | 0.124047082 | 0.752052236 |
|  | Valine | -0.591523254 | 0.124281362 | 0.752052236 |
|  | Arginine | 0.448436331 | 0.131285345 | 0.752052236 |
|  | Guanidoacetic Acid | -0.083725864 | 0.141392958 | 0.752052236 |
|  | Beta-Guanidinopropionic Acid | -0.7055468 | 0.167715975 | 0.752052236 |
|  | 3-Phosphonooxypyruvate | -0.698202981 | 0.179390338 | 0.752052236 |
|  | Alanine | -0.420834832 | 0.181669522 | 0.752052236 |
|  | Gamma-Aminobutyric Acid (GABA) | -0.389490149 | 0.720494594 | 0.94374644 |
|  | | | | |
| Prostaglandin formation from arachidonate | Arachidonic Acid | -0.003185279 | 0.131285345 | 0.752052236 |
|  | Anandamide | -0.413086737 | 0.126959983 | 0.752052236 |
|  | Glutathione Reduced | -0.71361459 | 0.01729343 | 0.737411281 |
|  | Ascorbate | -2.955501619 | 0.049829029 | 0.752052236 |

**Supplemental Table I.** Differentially expressed metabolites between NRP + SCS 2h and NRP + HOPE 24 h hearts associated to the relative functional pathways.
