# Supplementary figures and images for: Molecular and cellular adaptations to extended hypothermic oxygenated perfusion in donation-after-circulatory-death hearts in a porcine model
Source: Front Cardiovasc Med. 2026 May 25;13:1800470. doi: 10.3389/fcvm.2026.1800470 (PMC13243425; doi:10.3389/fcvm.2026.1800470)

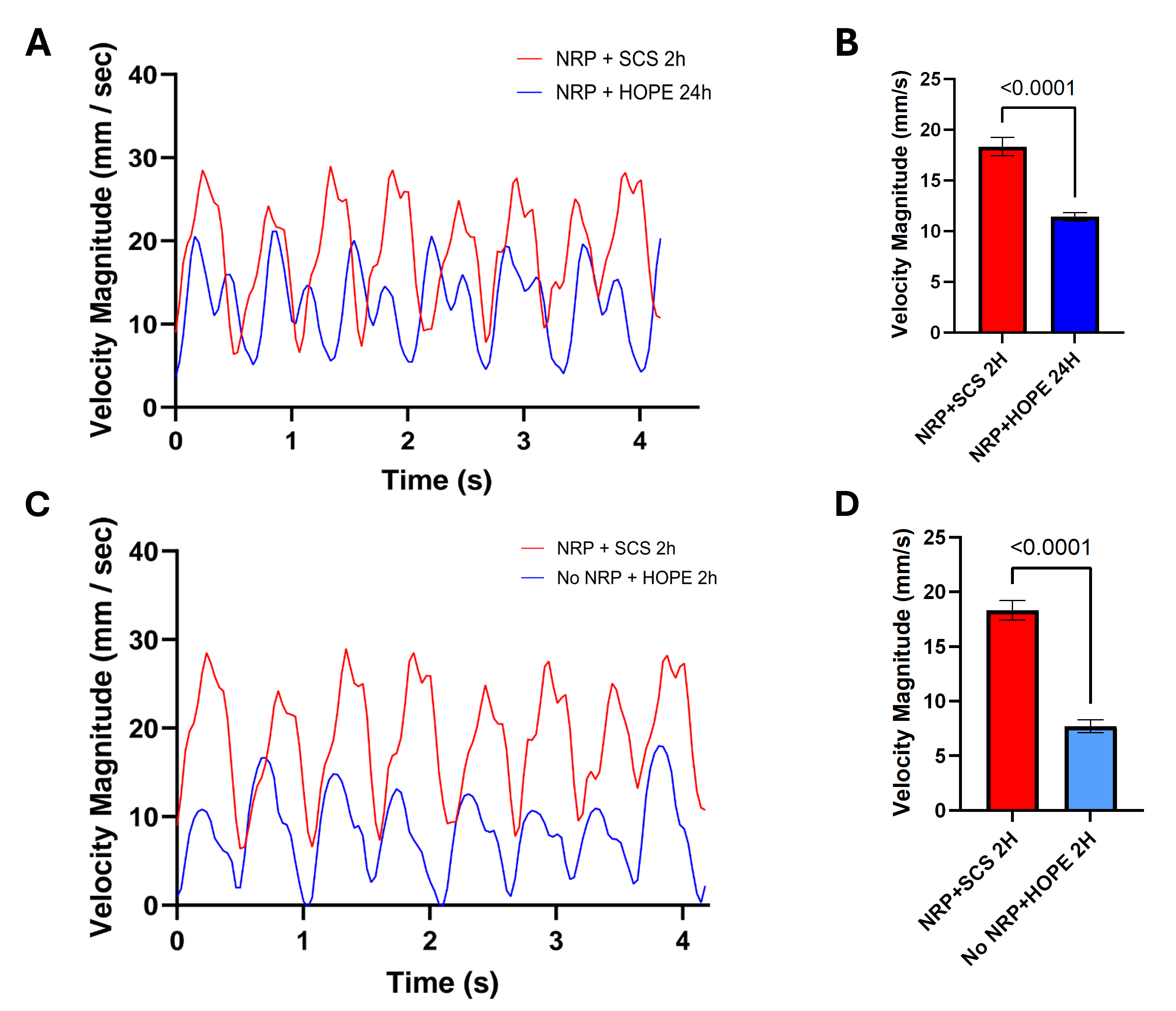

Supplement: Supplementary Figure S1 — Myocardial motion video quantification. Velocity traces during bench-top reperfusion show greater contractile amplitude in NRP+SCS 2 h vs NRP+HOPE 24 h (A) and vs No NRP+HOPE 2 h (C). Quantification of average velocity magnitude (mm/s) ± SEM confirmed higher contractile activity in NRP+SCS 2 h for both comparisons (p < 0.0001 by unpaired two-tailed Student's t-test followed by two-tailed Mann–Whitney U test) (B and D). [file Image1.tif]

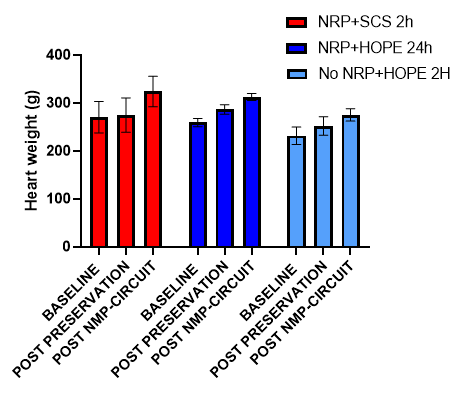

Supplement: Supplementary Figure S2 — Heart weight changes during preservation and reperfusion. Heart weights were measured at baseline, after preservation, and following 2 h of normothermic machine perfusion (NMP). Both NRP+SCS (2 h) and NRP+HOPE (24 h) hearts showed comparable total weight gain (∼20%), while No NRP+HOPE (2 h) hearts exhibited a smaller increase (∼15%). Data are shown as mean ± SEM. Two-way Anova analysis did not show any statistically significant differences. [file Image2.tif]

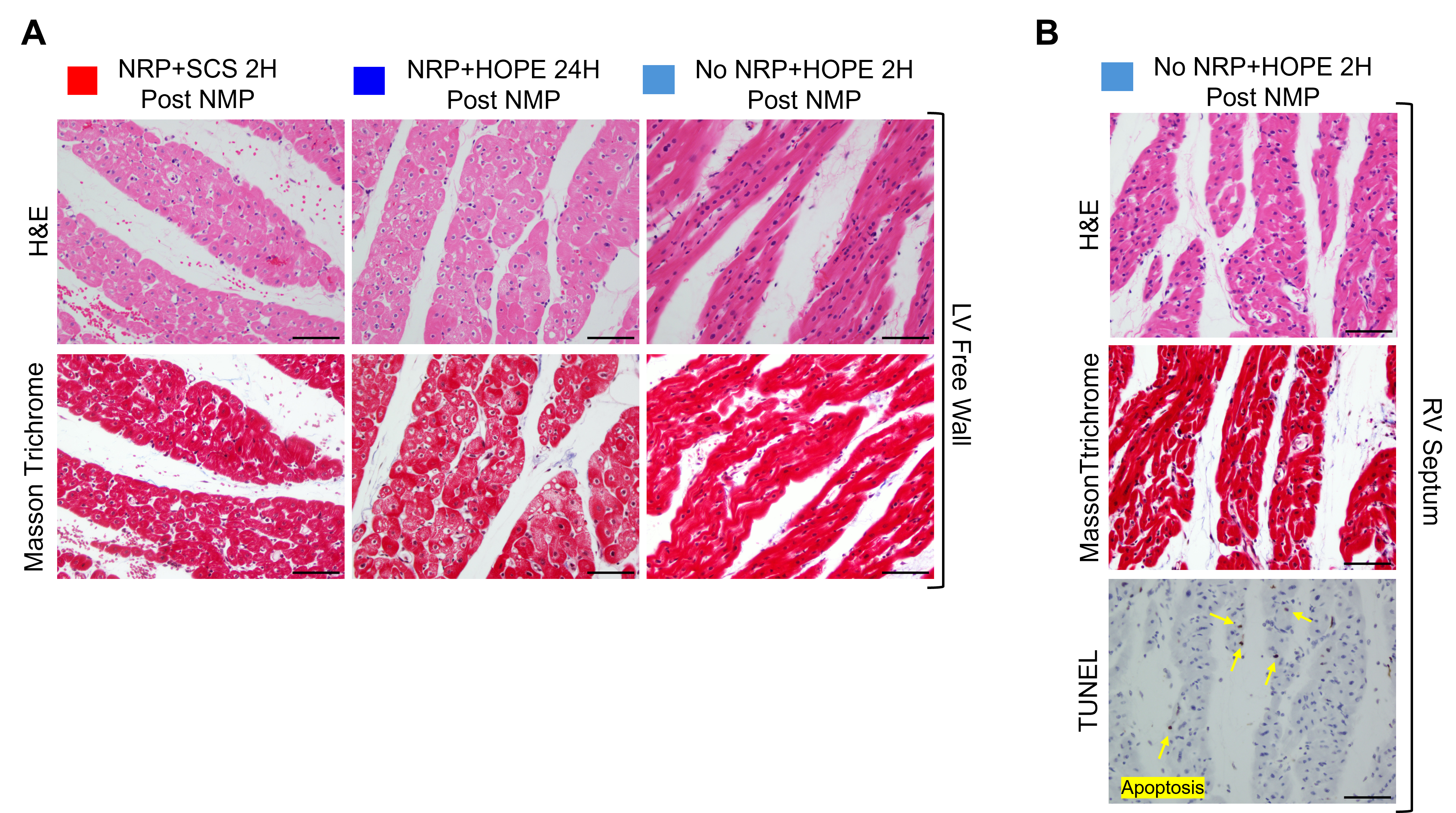

Supplement: Supplementary Figure S3 — Histological analysis of cardiac tissue. (A) Hematoxylin and Eosin (up) and Masson Trichrome(down) staining of the Left Ventricular Free Wall obtained by endomyocardial biopsies. (B) H&E, Masson Trichrome and TUNEL staining of RV Septum endomyocardial biopsies obtained from a No NRP heart preserved in HOPE for 2 h. Scale bar: 100 µm. [file Image3.tif]

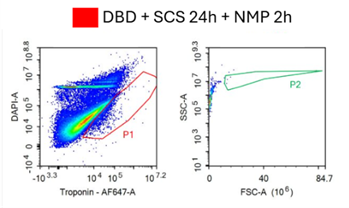

Supplement: Supplementary Figure S4 — Cardiomyocyte integrity after extended SCS preservation in a model of Donation following Brain Death (DBD). (A) Representative flow cytometry plots of DAPI and troponin staining in isolated cardiomyocytes after DBD procurement followed by 24 h SCS and 2 h of NMP reanimation circuit. Cardiomyocytes were identified as DAPI-positive, cardiac troponin T- positive events (P1 gate) and further selected for intact cells in a FSC vs SSC plot (P2 gate). The population of isolated intact cardiomyocytes was severely depleted, with no preserved cellular viability following 24 h of SCS, despite the more favorable donation model (20). [file Image4.tif]

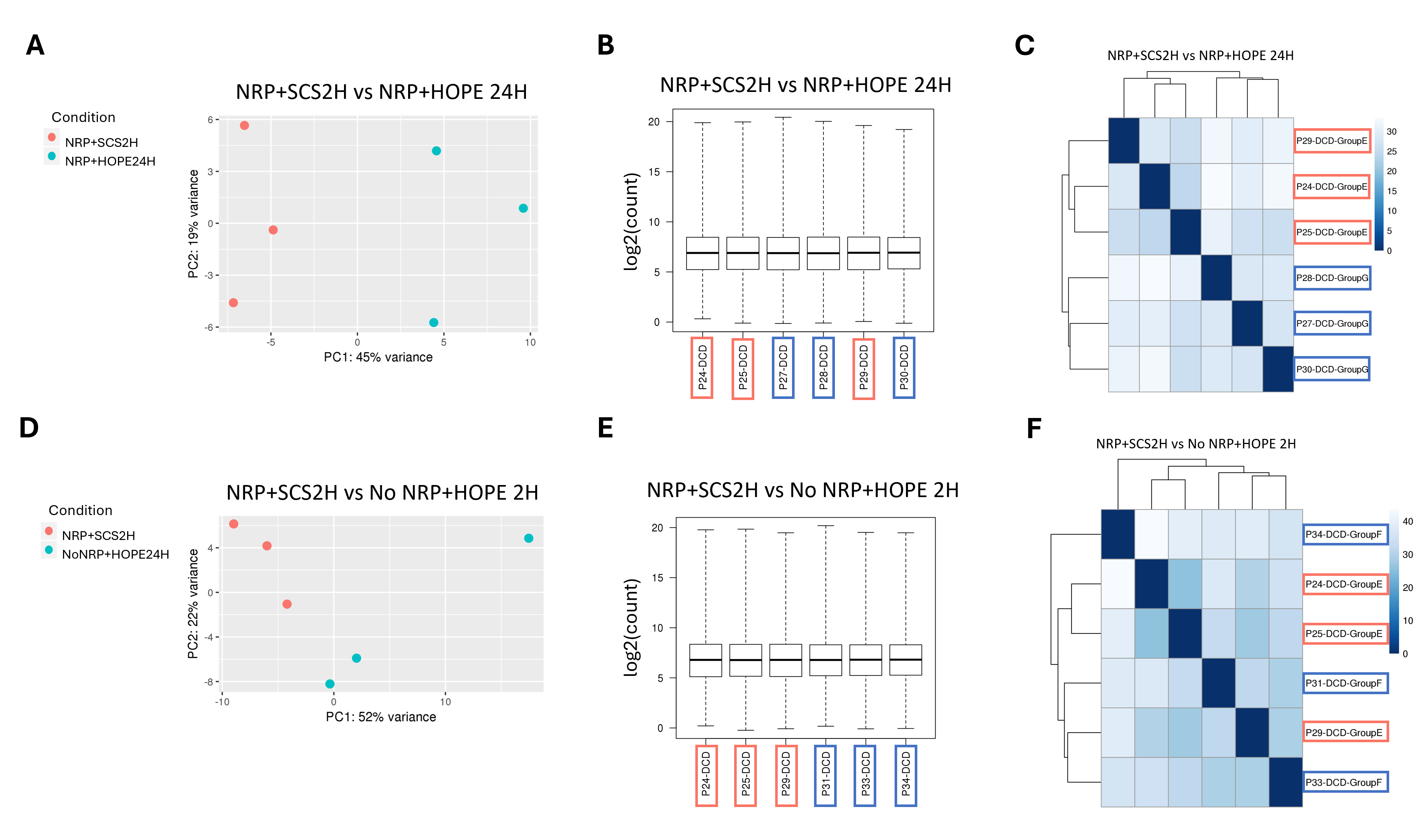

Supplement: Supplementary Figure S5 — Transcriptomic profiles of DCD hearts preserved with NRP+SCS 2 h versus HOPE 24 h or No NRP+HOPE 2 h. (A–C) Comparison of NRP+SCS 2 h and NRP+HOPE 24 h (A) Principal component analysis (PCA) shows partial separation between groups. (B) Boxplots of normalized read counts illustrate similar global expression distributions across samples. (C) Unsupervised hierarchical clustering heatmap of the top differentially expressed genes demonstrates partial grouping by preservation strategy. (D–F) Comparison of NRP+SCS 2 h and No NRP+HOPE 2 h. (D) PCA again shows modest separation between groups. (E) Boxplots of normalized read counts show comparable expression distributions. (F) Unsupervised clustering heatmap reveals overlap between groups. [file Image5.tif]
