# Supplementary material for: Molecular and cellular adaptations to extended hypothermic oxygenated perfusion in donation-after-circulatory-death hearts in a porcine model
Source: Front Cardiovasc Med. 2026 May 25;13:1800470. doi: 10.3389/fcvm.2026.1800470 (PMC13243425; doi:10.3389/fcvm.2026.1800470)
Supplement: Supplementary file 14 [file Datasheet1.docx]

**Supplemental Materials and Methods**

*Simulating DCD Procurement model:* The swine donor underwent anesthesia induction with intramuscular Telazol (6 mg/kg), propofol (1 mg/kg), and buprenorphine (0.01-0.03 mg/kg). Animals were endotracheally intubated in the prone position and rotated to a supine position for mechanical ventilation and surgery. Anesthesia and analgesia were maintained intraoperatively with inhaled isoflurane and/or continuous infusions of midazolam (0.2 mg/kg/hr) and propofol (3-5 mg/kg/hr). A midline sternotomy was performed with a bone saw, and the heart and great vessels were exposed. Intravenous heparin (300 IU/kg) was given for systemic anticoagulation. The right atrium was cannulated with a single-stage 24 Fr venous cannula (DLP Medtronic, Minneapolis, MN). The distal ascending aorta was cannulated with a one-piece 18 Fr arterial cannula (EOPA Medtronic, Minneapolis, MN). The aortic root was cannulated with an antegrade cardioplegia cannula (Surge Cardiovascular, Grand Rapids, MI).

*Withdrawal phase for circulatory arrest:* To replicate procurement following circulatory death criteria, the pigs (n=6) were first confirmed as deeply sedated and administered intravenous vecuronium 0.6mg/kg bolus followed by infusion at rate of 0.6mg/kg/hr. After achievement of chemical paralysis, the donor swine vitals were closely monitored until a MAP less than 50 mmHg was established; this was noted as functional warm ischemia time. When pulsatile measurements completely diminished from the arterial line, circulatory arrest was confirmed by asystole on the electrocardiogram. This began a 15-minute no touch period, after which, NRP was initiated using a CPB circuit. Cardiac function gradually resumed, and hearts which fibrillated were electrically defibrillated. All hearts regained sinus rhythm and weaned off cardiopulmonary bypass. After one hour of reperfusion, hearts were arrested in a standard brain-dead fashion. The left atrial appendage and IVC were opened to vent the heart, the aorta was cross clamped proximal to the aortic cannulation site, and the hearts were arrested via antegrade delivery of two 1L bags of XVIVO Heart Solution cardioplegia, 1ml/L Heart Solution Supplement, and 25mmol/L sodium bicarbonate (McKesson #239985) at 4 °C. Sterile slush was applied during arrest, and a standard cardiectomy was performed. To assess the role of NRP, circulatory death parameters were standardized to group 1, however the hearts were procured by direct procurement (n=3) without NRP. In group-2, the aorta was cross-clamped following 15 minutes of warm ischemic time and the hearts were arrested using the same supplemented solution as group-1. Standard cardiectomies were performed, and the hearts were weighed at baseline for all groups.

*Preservation*: The hearts selected for SCS clinical control (n=3) were stored in 1L of XVIVO Heart Solution at 4 °C for 2 hours. Hearts randomly selected for HOPE preservation were stored in the XVIVO Heart Assist Transport (XVIVO Group, Gothenburg, Sweden) for 2 hours (n=3) or 24 hours (n=3). HOPE provides oxygenated perfusion using carbogen gas (95% O2, 5% CO2) at 8 °C. The heart preservation device was primed with XVIVO cardioplegic solution supplemented with 350ml packed red blood cells, 3mL of XVIVO Heart Solution Supplement, 20 IU insulin (Humulin ELN-1325240), 50 mg Imipenem and Cilastatin (WG Critical Care 44567–705–10 , 5000 IU unfractionated heparin (Sagent Pharmaceuticals 25021-0400-30) and 10 mmol Potassium (Hospira  00409-6635-01). The hearts are connected to the device through an aortic cannula and the tubing is deaired. A silastic shunt is fastened across the mitral valve to vent the left ventricle, preventing overdistention. Perfusion within the device was carried out at an aortic root pressure of 20 mmHg for 2 hours or 24 hours. Heart weight was measured at the end of preservation for all groups.

*Bench-Top Normothermic Machine Perfusion:* At the conclusion of preservation, nine hearts (n=9) were reanimated on a bench-top NMP circuit for 2 hours to induce ischemic reperfusion injury and assess gross cardiac rhythm and contractility by simulating reperfusion in unloaded conditions. Our NMP circuit consisted of a venous reservoir with Capiox FX05 oxygenator (Terumo Cardiovascular, Ann Arbor, MI), 3T Heater-Cooler system (LivaNova, London, UK), and Quantum 4-in. roller pump (Spectrum Medical, Gloucester, UK), and the circuit was primed with 500cc of whole blood from the donor pig and roughly 1000-2000 IU unfractionated heparin. The hearts were loaded onto the circuit through canulation of the ascending aorta (8Fr Bio-Medicus NextGen Pediatric Arterial Cannula, Medtronic, Minneapolis, MN) using the Seldinger technique. The left ventricle was vented through a silastic tube positioned across the mitral valve; this allows blood to drain into the venous return. The atria were widely opened, and the return from the heart was drained into a collecting reservoir. The circuit was slowly initiated by advancing the arterial line, the aorta was de-aired, and an aortic cross-clamp was applied to begin 2 hours of normothermic reperfusion. Hearts were paced at 100bpm by electrodes on the anterior epicardium of both ventricles.

*Myocardial Motion Video Quantification:* A custom Python algorithm was developed to quantify myocardial motion during NMP circuit reanimation. First, a pixel-to-centimeter scale factor was determined by manually selecting two points 1 cm apart on a representative video frame. This allows for a flow magnitude vector to be converted into arbitrary units. Next, a rectangular region of interest (ROI) was defined in each video to capture the maximal beating area on the anterior surface of the heart ventricles. To reduce artifacts of camera movement, videos were stabilized using dense optical flow, where median displacement vectors were applied to generate affine transformations that reduced global motion. Within the stabilized ROI, dense optical flow was computed frame-to-frame to estimate myocardial velocity. This quantity was converted to millimeters per second. The velocity signal was smoothed using a Savitzky-Golay filter to suppress noise while preserving physiological features. Systolic and diastolic phases were tracked, and displacement was calculated by integrating velocity across valid cardiac cycles.

*Histological analysis.* Endomyocardial biopsies were collected from the right ventricular (RV) septum and left ventricular (LV) free wall using an 8-mm biopsy punch at the end of two hours of NMP. Additionally, aortic valve leaflets, mitral valve leaflets and aortic root were collected. Tissues were fixed in 10% formalin for 24 hours, then transferred to 70% ethanol solution and embedded in paraffin. 5µm-thick sections were cut using a microtome and mounted on high-adherence glass microscope slides. Sections were stained with hematoxylin and eosin (H&E) and Masson’s trichrome and imaged on a Leica 10X microscope. Standard TUNEL staining was also performed to identify DNA fragmentation and evaluate cell apoptosis. Histological slides were evaluated across all groups for presence of coagulative myocyte necrosis (CMN), inflammation, edema, interstitial hemorrhage, fibrosis, thrombosis, and apoptosis.

*Flow Cytometry.*  RV septum biopsies (0.1 g) were obtained using an 8mm surgical punch and washed in PBS 1x (Corning). Tissues were then minced into ~2mm^3^ chunks, transferred to a warm enzymatic solution of DMEM (Gibco) with 450 U/ml Collagenase I (Worthington, LS004196), 60 U/ml DNase I (Millipore, DN25) and 60 U/ml hyaluronidase (Worthington, LS002592), and incubated in a rotating mixer (~65/70 rpm) at 37 °C for 1 hour. The enzymatic digestion was stopped with HBB buffer (2% HI FBS (Gibco) and 0.2% BSA (Prometheus) in Hanks′ Balanced Salt solution (Sigma, H9269) and the solution was strained through a 100μm strainer. The suspended cells were centrifuged at 350g for 5 min at 4 °C and the pellet was resuspended in ACK lysing buffer (Gibco, A10492) to lyse red blood cells. After 5 min of incubation at room temperature, cells were washed with 9 ml of DMEM (Gibco) and centrifuged at 350g for 5 min at 4 °C. Then, cells were incubated for 10 min on ice in blocking solution (PBS + 0.5% BSA), followed by a wash in PBS1x. Cells were fixed in 2% PFA for 20 min on ice and washed twice in PBS1x. After fixation, cells were permeabilized with 0.1% Triton in PBS 1x 0.5% BSA for 20 min at 4°C. Cells were stained with Alexa Fluor 647 mouse anti-cardiac troponin T (BD Bioscience – 565744) 1:50 for 1h on ice in blocking solution. DAPI (Invitrogen) was added (1:3000) and cells were analyzed with the NovoCyte Penteon Flow Cytometer Systems 5 Lasers (Agilent). Data were analyzed with NovoExpress 1.6.2 software. Detected events were gated to distinguish intact cardiomyocytes from debris. Specifically, cardiomyocytes were identified by first gating for DAPI and anti-cardiac troponin T staining; the selected population was then gated by SSC-A and FSC-A to separate the intact cells from troponin-positive debris and small apoptotic bodies, thus reducing the incidence of false positive events. Cardiomyocyte size and troponin expression were used as indicators of preserved cellular integrity. The flow cytometry analysis was performed at the Columbia Stem Cell Initiative Flow Cytometry core facility at Columbia University Irving Medical Center.

*RNA Extraction and Bulk RNA sequencing Analysis.*  RNA was extracted from apex of the hearts at the end of NMP reperfusion, flash frozen and subsequentially purified using RNeasy Mini Kit (Qiagen, 74106) as per manufacturer guidelines. Purified RNA samples were processed and sequenced by Azenta Life Sciences (Burlington, MA, USA). RNA samples were quantified using Qubit 3.0 Fluorometer (Life Technologies, Carlsbad, CA, USA) and RNA integrity was checked using Agilent TapeStation 4200 (Agilent Technologies, Palo Alto, CA, USA). Samples with RIN score > 6 were selected for library preparation. ERCC RNA Spike-In Mix (Cat: #4456740) from ThermoFisher Scientific, was added to normalized total RNA prior to library preparation following manufacturer’s protocol. RNA sequencing libraries were prepared using the NEBNext Ultra II RNA Library Prep Kit for Illumina using manufacturer’s instructions (NEB, Ipswich, MA, USA). RNA inputs were normalized to 100ng. Briefly, mRNAs were initially enriched with Oligod(T) beads. Enriched mRNAs were fragmented for 15 minutes at 94 °C. cDNA fragments were end-repaired and adenylated at 3’ends, and universal adapters were ligated to cDNA fragments, followed by index addition and library enrichment by PCR with limited cycles. The sequencing library was validated on the Agilent TapeStation (Agilent Technologies, Palo Alto, CA, USA), and quantified by using Qubit 3.0 Fluorometer (Invitrogen, Carlsbad, CA) as well as by quantitative PCR (KAPA Biosystems, Wilmington, MA, USA). The sequencing libraries were clustered on a flowcell. After clustering, the flowcell was loaded on the Illumina NovaSeq instrument according to manufacturer’s instructions. The samples were sequenced using a 2x150bp Paired End (PE) configuration. Image analysis and base calling were conducted by the Control software. Raw sequence data (.bcl files) generated by the sequencer were converted into fastq files and de-multiplexed using Illumina's bcl2fastq 2.17 software. One mismatch was allowed for index sequence identification.

After investigating the quality of the raw data, sequence reads were trimmed to remove possible adapter sequences and nucleotides with poor quality. The trimmed reads were mapped to the *Sus Scrofa* reference genome available on ENSEMBL using the STAR aligner v.2.5.2b. The STAR aligner is a splice aligner that detects splice junctions and incorporates them to help align the entire read sequences. BAM files were generated as a result of this step. Unique gene hit counts were calculated by using feature Counts from the Subread package v.1.5.2. Only unique reads that fell within exon regions were counted.

After extraction of gene hit counts, the gene hit counts table was used for downstream differential expression analysis. Using DESeq2, a comparison of gene expression between the samples was performed. The Wald test was used to generate p-values and log_2_ fold changes. The Benjamini–Hochberg correction was applied to generate adjusted p-values. Genes with an adjusted p-value < 0.05 and absolute log_2_ fold change value > 1 were called as differentially expressed genes for each comparison and visualized in volcano plots. Heatmaps of DEGs were generated using DataMap (v0.11). KEGG pathways were analyzed on mapped genes, and significant results (FDR < 0.01) were visualized using ShinyGO (v0.77).

*Untargeted Metabolomic Profiling.* Untargeted Metabolomic profiling was conducted using ultrahigh performance liquid chromatography-high resolution accurate mass spectrometry (HRAMS)^25^ . Metabolites were extracted from left ventricular biopsies obtained at the end of NMP reperfusion and immediately flash frozen. Samples underwent a two-step homogenization in 1:3 methanol/water (stable isotope labeled with internal standards) and 1:1 methanol/acetonitrile (5 µL mg⁻¹ tissue each). Ten microliters (10μL) of this combined extract after centrifugation was analyzed using a high-resolution accurate-mass (HRAM) platform consisting of Vanquish™ Duo UHPLC system equipped with dual split sampler configuration coupled to a Exploris 240 HRAM mass spectrometer (Thermo Fisher Scientific, San Jose, CA, USA).

Chromatographic separation was performed in triplicate using HILIC chromatography under positive ion mode and RP chromatography under negative ion mode, both at 60 °C. HILIC separation was done on a Waters XBridge BEH Amide XP column (2.1 x 50 mm, 2.5 μm) and gradient elution with mobile phases 0.2% formic acid in water (A) and acetonitrile (B). The initial 75% B at 0.35 mL/min kept for 1.5min, decreased linearly to 20% B at 4 min with a flow rate increase to 0.4 mL/min, and a final hold of 1 min. Reverse-phase separation was performed on a C18 column (Higgins Analytical Targa C18 2.1 × 50 mm, 3 μm) with 1mM Ammonium acetate in water (A) and acetonitrile (B) as mobile phases. The initial 35% B at 0.4 mL/min, was increased linearly to 95% B at 1.5 min and held for the remaining 3.5 min with a flow rate 0.5 mL/min. The HRMS was operated in a Full Scan (120,000 resolution) acquisition mode and a data dependent MS2 (ddMS2) acquisition mode at resolutions of 60,000 (full scan) and 15,000 (ddMS2 scan), both positive and negative polarity mode to acquire the spectral data. The HRMS source parameters were as follows: spray voltage 3.5 kV (+ESI), 3.00 KV (-ESI); capillary temperature 300 ℃; sheath gas flow rate 45; Aux gas 25 AU (+ESI), 15 AU (-ESI); sweep gas flow rate 1 AU; Aux gas heater temperature 250 ℃.

The raw data files acquired were processed through a computational pipeline that leverages open-source R packages – Asari software^26^ and xMSanalyzer. Asari uses Gaussian model with area under the curve (auc) method for peak area calculation, 10 seconds maximum shift, 5 ppm mass tolerance, signal to noise ratio as 3:1, lowess algorithm for retention time alignment, retention time threshold as 20 seconds and minimum peak intensity of 1e6 to create feature table. Metabolite features were filtered by 50% presence in a determined group. Data were log2 transformed (to stabilize variance), and missing values were imputed using half of the minimum intensity for each feature.

Feature annotation was achieved by an in-house reference library as well as HMDB, KEGG, LipidMAPS as databases. Multivariate (PCA) and univariate (t-test) were performed using R to visualize sample clustering and identify features contributing to group separation. For each identified metabolite, the null hypothesis that there were no differences in area means across the groups was tested using Student’s t-test. Correction for the comparisons was performed using the Benjamini and Hochberg FDR method. The adjusted p-values (q-values) reflect correction for the total number of metabolites tested. Significant metabolites were identified using an absolute log₂ fold-change cutoff of 0.693 (1.6-fold) in combination with an adjusted p-value threshold of 0.05.

Metabolic pathway functional and enrichment analysis of the detected features were performed using MetaboAnalyst's mummichog algorithm using the KEGG metabolic pathway databases as the reference databases. Pathways with a p-value < 0.05 were considered statistically significant.
